# Does Targeting CPP at CPPopt Actually Improve Cerebrovascular Reactivity? A Secondary Analysis of the COGiTATE Randomized Controlled Trial

**DOI:** 10.1007/s12028-024-02168-y

**Published:** 2024-12-02

**Authors:** Erta Beqiri, Jeanette Tas, Marek Czosnyka, Ruud C. R. van Kaam, Joseph Donnelly, Roel H. Haeren, Iwan C. C. van der Horst, Peter J. Hutchinson, Sander M. J. van Kuijk, Annalisa L. Liberti, David K. Menon, Cornelia W. E. Hoedemaekers, Bart Depreitere, Geert Meyfroidt, Ari Ercole, Marcel J. H. Aries, Peter Smielewski

**Affiliations:** 1 Brain Physics Laboratory, Department of Clinical Neurosciences, Division of Neurosurgery, University of Cambridge, Cambridge, UK; 2 Department of Neurology, Kepler University Hospital, Johannes Kepler University Linz, Linz, Austria; 3 Research Institute for Neuroscience, Johannes Kepler University Linz, Linz, Austria; 4 Department of Intensive Care Medicine, University Maastricht, Maastricht University Center Maastricht, Maastricht, The Netherlands; 5 Department of Intensive Care Medicine, Radboud University Medical Center, Nijmegen, The Netherlands; 6 Department of Neurosurgery, University Maastricht, Maastricht University Center Maastricht, Maastricht, The Netherlands; 7 School for Mental Health and Neuroscience, University Maastricht, Maastricht, The Netherlands; 8 Cardiovascular Research Institute Maastricht, Maastricht, The Netherlands; 9 Division of Neurosurgery, Addenbrooke’s Hospital and University of Cambridge, Cambridge, UK; 10 Department of Clinical Epidemiology and Medical Technology Assessment, Maastricht University Medical Center+, Maastricht, The Netherlands; 11 Department of Anaesthesia and Intensive Care, San Carlo Borromeo Hospital, Milan, Italy; 12 University Division of Anaesthesia, University of Cambridge, Addenbrooke’s Hospital, Cambridge, UK; 13 Department of Neurosurgery, University Hospital Gasthuisberg, Katholieke Universiteit Leuven, Louvain, Belgium; 14 Department and Laboratory of Intensive Care Medicine, Katholieke Universiteit Leuven, Louvain, Belgium

**Keywords:** Cerebral autoregulation, Pressure reactivity index, Cerebral perfusion, Intensive care, Optimal cerebral perfusion pressure, Optimal pressure reactivity, Precision medicine, Traumatic brain injury

## Abstract

**Background:**

The 'CPPopt-Guided Therapy: Assessment of Target Effectiveness' (COGiTATE) randomised controlled trial demonstrated the feasibility and safety of targeting an automated cerebral perfusion pressure (CPP) tailored to optimize cerebrovascular autoregulation (CPPopt) in patients with traumatic brain injury (TBI) requiring intracranial pressure management. The average values of the autoregulation index known as the pressure reactivity index (PRx) were not different between the intervention (CPP target = CPPopt) and control (CPP target = 60–70 mmHg) groups of the trial. This secondary analysis was performed to investigate whether: (1) in the intervention group, PRx was closer to PRxopt (PRx at CPPopt) values, indicating a more preserved reactivity, as opposed to in the control group; (2) in the intervention group, patients experienced lower hourly PRx when CPP was close to the CPPopt-based target.

**Methods:**

We analyzed data from the 28 and 32 patients randomized to the control and intervention groups of the COGiTATE study, respectively. We compared hourly averaged ΔPRx (PRx minus PRxopt, where PRxopt is PRx at CPPopt) between the two groups, focusing on periods of globally preserved/homogeneous autoregulation (negative PRxopt). For each patient in the intervention group, PRx values in periods when ΔCPP (CPP minus CPPopt target) was between −5 and + 5 mm Hg were compared to values in periods when ΔCPP was outside this range.

**Results:**

The median ΔPRx was significantly lower in the intervention group for negative PRxopt (Mann–Whitney *U*-test, *p* < 0.001). For each patient in this group, the median PRx was lower in periods when CPP was close to the CPPopt-based target (Wilcoxon test, *p* < 0.001).

**Conclusions:**

Despite no statistically significant difference in the grand mean PRx, our results suggest that targeting CPPopt does provide a way of improving cerebrovascular reactivity in patients with TBI, offering a rational intervention for trials that address this issue. We also bring insight into aspects of the PRx/CPP relationship that should be considered for autoregulation-guided management for future clinical protocols and trials design.

## Introduction

Cerebral perfusion targets set according to cerebrovascular reactivity have been suggested and investigated as one of the strategies for individualized management in patients with traumatic brain injury (TBI) admitted to critical care [1–3]. Although extensive retrospective research has been conducted over the past 20 years, the prospective evaluation of such strategies is at its infancy [3]. As a consequence, the physiological effect of targeting cerebral perfusion pressure (CPP) at the optimal cerebral perfusion pressure (CPPopt) tailored to optimize cerebrovascular reactivity, as a surrogate for cerebral autoregulation, is still unknown.

The hypothesis behind autoregulation-guided management is that by optimizing cerebral autoregulation, episodes of brain hypoperfusion or hyperemia are avoided, as the brain is maintained at a CPP where it can best maintain adequate cerebral blood flow in the face of systemic changes [4]. This should translate into better clinical outcome by limiting secondary injury [5, 6]. Therefore, targeting CPP at the level at which autoregulation is best preserved should translate into achieving the best autoregulation possible. The index of autoregulation used for evaluating CPPopt is the pressure reactivity index (PRx) [7] (Supplementary Material 1). It is logical to postulate that PRx should indicate a better preserved vascular reactivity if CPP is at CPPopt levels.

To date, the only concluded prospective interventional trial on autoregulation-guided management in patients with TBI is the CPPopt-Guided Therapy: Assessment of Target Effectiveness (COGiTATE) trial [8], which showed that targeting CPP at CPPopt is feasible and safe in patients with TBI requiring intracranial pressure management (NCT02982122, www.clinicaltrials.gov). In the intervention group of the study, CPP targets were adjusted every four hours according to an automated estimation of CPPopt [9], assessed via customized ICM + software at the bedside. In the control group, CPP was targeted between 60 and 70 mmHg, according to the Brain Trauma Foundation guidelines. Although the trial was not powered for effectiveness, it was notable that the grand mean value of PRx was not significantly different between the control and intervention group of the COGiTATE trial (mean 0.0331 [SD 0.199] vs. –0.0417 [0.231], *p* = 0.2, *t*-test). While this might seem unexpected, there are several reasons that could explain such result beyond the power of the study. In this paper we explore the behavior of PRx at different granularity in the COGiTATE cohort, comparing control and intervention groups. We hypothesize that in the intervention group, PRx was closer to PRxopt (PRx at the level of CPPopt) values, indicating a more preserved reactivity, as opposed to in the control group.

As a further investigation, we considered the intervention group separately and investigated the difference in PRx values, comparing periods with CPP within the autoregulation target versus periods with CPP outside the autoregulation target, for individual patients. We hypothesize that in the intervention group, patients experienced a better preserved vascular reactivity when CPP was within the CPPopt-based target.

## Methods

We performed a secondary analysis [10] on the COGiTATE prospectively collected data.

### Trial Design

The protocol of the trial [11], the data collection, and the main results of the study [8] have been described elsewhere. Briefly, the COGiTATE trial was a prospective multicenter non-blinded randomized control phase II study in which patients with TBI requiring intracranial pressure (ICP)–directed therapy were randomized into a control group or intervention group. Six times per day, a software-based bedside alert would prompt the clinical team to review the CPP target for the next hours according to a recommended CPP target. In the control group, the CPP target was within the Brain Trauma Foundation guidelines range (CPP 60–70 mm Hg) [1]. In the intervention group, the CPP target was based on an automated estimation of CPPopt [9, 11].

### Physiological Rationale for the Secondary Analysis

In the next paragraphs, we provide the physiological rationale behind our investigation into the reasons for the apparent lack of a significant difference in the grand mean PRx between the control and intervention groups, beyond the original study’s inadequate power to assess physiological outcomes.

**Fig. 1:**
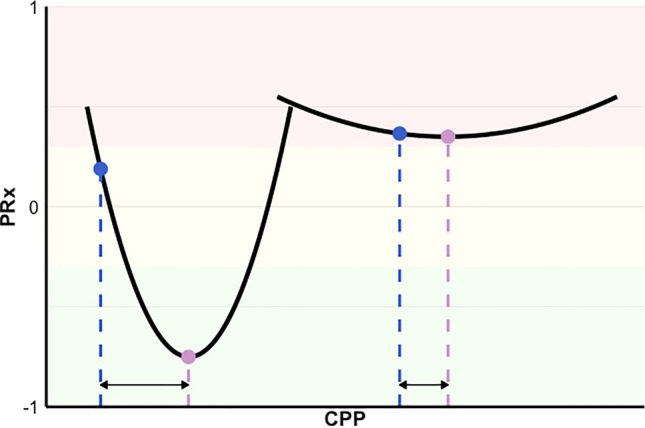
Conceptual scheme for location and shape of the U-shape curve. The figure is a schematic representation for illustrating basic concepts related to location and shape of the curve generated from the relationship between the pressure reactivity index (PRx) and cerebral perfusion pressure (CPP) over a certain amount of time. The left curve has a large y-span, ranging from PRx values that indicate impaired vascular reactivity (red shaded area in the plot) to PRx values that indicated preserved reactivity (green area). On the contrary, the right curve is almost flat, denoting that PRx indicates impaired vascular reactivity for any CPP value. In both cases, optimal CPP (CPPopt) can be identified as the CPP value corresponding to the lowest PRx (pink dot and dashed line). The hypothetical current CPP value of the patient (blue dot and dashed line) can be compared to CPPopt in both cases. In the right curve scenario, the difference between CPP and CPPopt is small (high compliance with the CPPopt target); however, the PRxopt (PRx corresponding to CPPopt) is in a range of impaired vascular reactivity. In the left curve scenario, the difference between CPP and CPPopt is much larger, but the PRx value at the current CPP is already at a lower level than the PRx value of the CPP of the right curve. In addition, in the left curve scenario, a higher compliance to the CPPopt target would allow for reaching levels of PRx at preserved vascular reactivity. Note that the figure is simplified for illustrative purposes. The curve shape and location can span through the whole range of both PRx and CPP; however, we did not describe here in detail other possible scenarios for the sake of simplicity. In our analysis, PRxopt was calculated via the automated multiwindow-based algorithm used in the trial for CPPopt assessment, and this algorithm has restrictive curve fitting and weighting criteria [9]

The relationship between CPP and PRx can in general be described as a U-shaped curve (clearly discernible in some percentage of cases) constrained to a PRx range (−1 to 1). The optimum value of this PRx/CPP curve is identified by an x-optimum (CPPopt) and a y-optimum (PRxopt). The latter can correspond to different levels of PRx, defining the vertical location of the curve within the plot. The shape of such a curve can also differ based on the y-span of the curve within the physiological CPP limits (see Fig. 1). If PRxopt is markedly positive, the curve has a small autoregulatory span, and the vascular reactivity would be impaired at any CPP level. As a consequence, targeting CPPopt would not allow to move from a non-autoregulating to an autoregulating physiology, but simply to maintain the best possible reactivity for that period. On the contrary, in cases when PRxopt is negative and the shape of the curve has a wide y-span (PRx ranges from negative to positive within the physiological CPP values), it is more plausible that targeting CPPopt would provide means for achieving a good vascular reactivity. A simple metric of deviation of CPP from CPPopt (ΔCPPopt) does not convey the difference between those two cases (see Fig. 1 for more details). Similarly, a simple comparison of the averages of PRx between patient groups would also very likely be heavily affected by those effects. These concepts lead to the formulation of the primary hypothesis.

Further, the feasibility results of the COGiTATE study showed that the percentage of time with patients’ CPP concordant to the CPPopt-based target value was on average 46.5% in the intervention group [8]. It is possible that this creates a dilution effect on the difference in PRx. This led to the formulation of the secondary hypothesis.

### Materials

All patients included in the main analysis of the trial are also included in the current analysis: 28 patients for the control group and 32 for the intervention group. The high-resolution monitoring data were considered in this analysis: we retrieved deidentified and cleaned minute-by-minute time trends of CPP, PRx, CPPopt, PRxopt, and the six times daily review results for the duration of the study. CPPopt and PRxopt were both calculated with the automated multiwindow approach adapted for prospective use in the COGiTATE study [9].

### Data Processing

ICM + software [12, 13] and Python v3.9 were used for further data processing of the minute-by-minute time trends. One-hour average values of the above variables were used for further analysis to increase the signal-to-noise ratio.

For the primary objective, we compared hourly averages of ΔPRx (where ΔPRx = PRx − PRxopt) between control and intervention groups for values of ΔPRx below subsequent values of PRxopt using steps of 0.05. We observed that PRxopt spanned from 0.60 to −0.90. However, below −0.75, the data were not represented for both groups; therefore, the comparison could not be performed. Furthermore, the hourly periods available below the threshold of −0.60 were less than ten periods, making them very scarcely represented. We excluded those periods from the analysis; hence, the lowest threshold shown in the results is −0.60.

For the secondary objective, we considered the data of patients included in the intervention group (*n* = 32). We calculated the deviation of CPP from the CPPopt-based target (CPPtarget) as CPP − CPPtarget for CPP above CPPtarget and as CPPtarget − CPP for CPP below CPPtarget. In this analysis, we considered all study periods, including the first hours after inclusion. For each patient, we defined the periods of “CPP within the target range” as the periods with the deviation of CPP from the CPPtarget between −5 and + 5 mm Hg. All the other periods were defined as “CPP outside the target range.” This distinction was based on the analysis performed to test the feasibility end point of the trial. For further statistical evaluation, we considered one average value for each type of period and for each patient. PRx was Fisher-transformed prior to obtaining the hourly average values [14].

### Statistical Analysis

Statistical analysis was performed in R software v 4.0 [15]. Normality of continuous variables was assessed with histograms and the Shapiro–Wilks test. Values of continuous variables are presented as median (1st quartile – 3rd quartile) accordingly. The comparisons were visualized with violin and box plots. For the primary objective, we performed between-groups comparison (control vs. intervention, Mann–Whitney *U*-test) of ΔPRx for PRxopt below different levels. The Bonferroni method was considered for adjusting for multiple comparisons. For the secondary objective, we performed a Wilcoxon test for patients in the intervention group, comparing PRx at periods of CPP within the target range as opposed to periods of CPP outside the target range. A *p* value < 0.05 was considered statistically significant.

## Results

The cohort demographics are described in Tas et al. [8] and are reported here in Table 1.

**Table 1:** Patients baseline characteristics

	CPP control (*n* = 28)	CPP intervention (*n* = 32)	Total (*n* = 60)
Sex, men, *n* (%)	21 (75)	22 (69)	43 (72)
Age, mean (SD), years	48 (19)	42 (17)	45 (18)
Initial assessed median GCS motor score (q1–q3)	4 (2–5)	4 (1–6)	4 (1–5)
Initial assessed GCS 3–8 category, *n* (%)	21 (75)	20 (63)	41 (68)
Initial assessed GCS 9–13 category, *n* (%)	6 (21)	7 (22)	13 (22)
Initial assessed GCS 14–15 category, *n* (%)	1 (4)	5 (16)	6 (10)
Pupil fixed and dilated,* n* (%)			
Unilateral	4 (14)	3 (9)	7 (12)
Bilateral	2 (7)	1 (3)	4 (7)
CT-Marshall classification, *n* (%)			
Diffuse injury (I)	0 (0)	1(3)	1 (2)
Diffuse injury (II)	17 (61)	21(66)	37 (63)
Diffuse injury (III)	6 (21)	2 (6)	8 (14)
Diffuse injury (IV)	3 (11)	0 (0)	3 (5)
Evacuated mass lesion (V)	0 (0)	4 (13)	4 (7)
Nonevacuated mass lesion (VI)	2 (7)	4 (13)	6 (10)
Isolated head injury, *n* (%)	9 (32)	17 (53)	26 (43)
Median IMPACT outcome (mortality) prediction (q1–q3)^a^	32 (25–46)	29.5 (15.0–34.5)	31.5 (23.5–39)

Presented percentages may not be equal to 100% as a result of rounding. Table adapted from Table 1 in Tas et al. [8]

CPP, cerebral perfusion pressure, CT, computed tomography, GCS, Glasgow Coma Scale, IMPACT, International Mission for Prognosis and Analysis of Clinical Trials in Traumatic Brain Injury, q1: 1st quartile, q3: 3rd quartile

^a^The IMPACT score for moderate to severe traumatic brain injury [20]. Included patient characteristics in the prognostic model for mortality prediction at 6 months using the core model: age, GCS motor score, and pupillary reactivity. IMPACT is validated for patients with traumatic brain injury with an initial GCS ≤ 12 (control *n* = 27, intervention *n* = 26). In addition, three patients with missing Glasgow Outcome Scale at 6 months (*n* = 1 in control group and *n* = 2 in intervention group) were not included for the average IMPACT outcome prediction scores

### ΔPRx for Different PRxopt Thresholds (Objective 1)

**Fig. 2:**
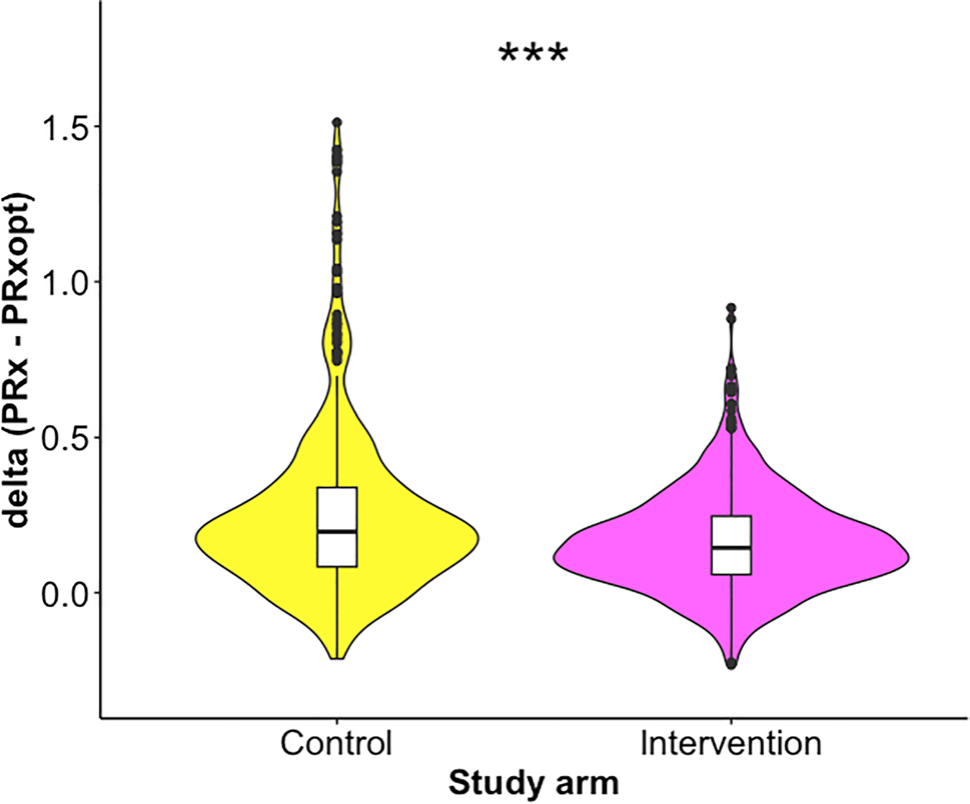
ΔPRx for PRxopt < −0.30 for the two study arms. Hourly data of average deviation of PRx from PRxopt were filtered for periods when PRxopt was below −0.30. The violin and box plots show the comparison between control (number of monitoring hours = 415, number of patients = 20) and intervention group (number of monitoring hours = 736, number of patients = 27) for these periods. This example is aimed to reflect the left curve case scenario described in Fig. 1. Table 2 summaries statistics for all different levels of PRxopt considered. ***p < 0.001. PRx pressure reactivity index; PRxopt optimal pressure reactivity index.

ΔPRx was available for a total number of 3355 monitoring hours across the cohort (out of the total number of 4374), with 1560 (47%) epochs in the control group and 1795 (53%) epochs in the intervention group. Table 2 shows the results of the comparison of ΔPRx for different levels of PRxopt and between control and intervention patients. ΔPRx was significantly lower in the intervention group for PRxopt below negative values. The difference is significant starting for PRxopt < −0.15 (not corrected for multiple comparisons), which occurred in 817 of 1560 (52%) epochs in the control group and in 1124 of 1795 (63%) epochs in the intervention group. Figure 2 visualizes an example of the comparison for PRxopt < −0.30 (arbitrary number) with violin and box plots.

**Table 2:** Comparison of ΔPRx between control and intervention groups at PRxopt below thresholds

	Control group			Intervention group			Mann–Whitney *U*-test	
PRxopt threshold	*n* monitoring hours (total = 1,560)	*n* patients (total = 28)	ΔPRx, median (q1–q3)	*n* monitoring hours (total = 1,795)	*n* patients (total = 32)	ΔPRx, median (q1–q3)	*p* value	*p* value adjusted
0.60	1560	28	0.106 (0.012–0.22)	1795	32	0.109 (0.015–0.217)	ns	ns
0.55	1552	28	0.106 (0.012–0.22)	1774	32	0.11 (0.017–0.217)	ns	ns
0.50	1546	27	0.106 (0.011–0.22)	1766	32	0.11 (0.019–0.218)	ns	ns
0.45	1542	27	0.105 (0.011–0.219)	1752	32	0.11 (0.019–0.217)	ns	ns
0.40	1537	27	0.105 (0.011–0.22)	1737	32	0.111 (0.02–0.217)	ns	ns
0.35	1529	27	0.106 (0.012–0.22)	1720	32	0.111 (0.02–0.217)	ns	ns
0.30	1505	27	0.106 (0.012–0.221)	1695	32	0.111 (0.02–0.216)	ns	ns
0.25	1492	27	0.107 (0.012–0.222)	1669	32	0.112 (0.022–0.219)	ns	ns
0.20	1457	27	0.109 (0.013–0.223)	1641	32	0.112 (0.023–0.219)	ns	ns
0.15	1414	27	0.112 (0.017–0.228)	1606	31	0.115 (0.024–0.219)	ns	ns
0.10	1352	26	0.116 (0.023–0.233)	1544	31	0.117 (0.028–0.22)	ns	ns
0.05	1280	26	0.123 (0.028–0.236)	1490	30	0.121 (0.031–0.221)	ns	ns
0.00	1179	26	0.128 (0.035–0.244)	1425	30	0.124 (0.034–0.222)	ns	ns
−0.05	1072	25	0.128 (0.034–0.245)	1347	30	0.128 (0.038–0.226)	ns	ns
−0.10	951	24	0.138 (0.044–0.262)	1254	30	0.131 (0.042–0.229)	ns	ns
−0.15	817	24	0.144 (0.049–0.272)	1124	29	0.134 (0.047–0.231)	< 0.05	ns
−0.20	690	23	0.154 (0.053–0.285)	1005	29	0.136 (0.049–0.231)	< 0.01	ns
−0.25	546	20	0.174 (0.068–0.307)	865	29	0.14 (0.055–0.239)	< 0.001	< 0.01
−0.30	415	20	0.196 (0.083–0.339)	736	27	0.144 (0.059–0.247)	< 0.001	< 0.001
−0.35	297	17	0.213 (0.097–0.372)	600	25	0.152 (0.067–0.247)	< 0.001	< 0.001
−0.40	191	12	0.244 (0.128–0.397)	467	23	0.156 (0.076–0.255)	< 0.001	< 0.001
−0.45	137	12	0.229 (0.121–0.38)	322	23	0.169 (0.083–0.28)	< 0.001	< 0.001
−0.50	92	10	0.225 (0.124–0.381)	194	17	0.176 (0.094–0.305)	< 0.01	ns
−0.55	39	9	0.358 (0.225–0.459)	115	11	0.193 (0.094–0.315)	< 0.001	< 0.001
−0.60	12	3	0.361 (0.299–0.473)	72	6	0.229 (0.106–0.34)	< 0.01	ns

For each PRxopt threshold, the data are filtered and only hourly periods with PRxopt below that threshold are considered. The number of total monitoring hours that satisfy the threshold as well as the number of patients uniquely represented are shown for each group and for each threshold. The Bonferroni method was used for adjusting for multiple comparisons

q1: 1st quartile; q3: 3rd quartile; ns, not significant; ΔPRx = PRx − PRxopt

### CPP Within Compared to CPP Outside the CPP Target Range and the Corresponding PRx Values (Objective 2)

**Fig. 3:**
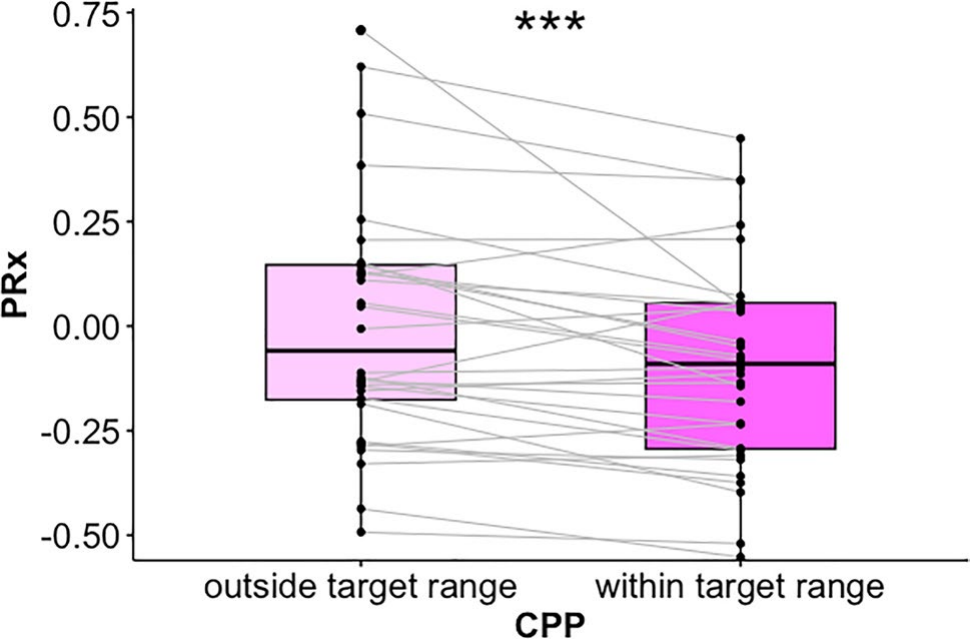
Difference in PRx values for CPP within or outside the target range. For each patient included in the intervention group, the average value of PRx (after Fisher transformation) for periods when CPP was outside the target range is compared with PRx for periods when CPP was inside the target range. ***p < 0.001. CPP cerebral perfusion pressure, PRx pressure reactivity index

Figure 3 illustrates the paired comparison for the secondary objective. For each patient in the intervention group, median PRx was lower in periods of CPP within the target range as opposed to periods of CPP outside the target range (−0.092 [−0.269 to −0.053] vs. −0.057 [− 0.157 to −0.123], respectively, *p* < 0.001).

## Discussion

In this secondary analysis, we retrospectively investigated the effect that optimizing CPP at CPPopt has on cerebrovascular reactivity in patients with TBI who require ICP-directed therapy. We demonstrated that it seems possible to improve vascular reactivity by targeting CPPopt, provided that the margin for improvement exists. We tested our hypothesis on data collected during the multicenter phase II randomized controlled trial on CPPopt-guided therapy, namely COGiTATE [8].

The TBI patients enrolled in the intervention group of the COGiTATE study represent a unique group of patients in whom dynamic CPPopt time trends were used for regular CPP target assessments over a maximum of first 5 days of admission in the critical care unit. The results of the trial showed that it was feasible to target CPP at CPPopt and that these patients spent on average 46.5% (41.2–58%) of time with CPP concordant with the CPPopt-based target. In the current study, we explored the effect that such target adherence had on brain physiology. Our secondary analysis showed that the vascular reactivity, as measured by PRx, was better preserved when CPP was within the target range for each patient. This may sound obvious, but one must note that the CPPopt value is a mathematical construct, a minimum of a parabolic curve fitted to Fisher-transformed PRx values. Therefore, the PRx value at the CPPopt is only the minimum of that smooth curve, potentially forcibly fitted into a relatively widely varied PRx/CPP relationship, possibly all staying in the entirely impaired or entirely preserved section of the plot. Thus, our result serves as a kind of “sanity check,” a proof of concept that aligns with the overall hypothesis behind autoregulation-guided management. It also suggests that increasing compliance with autoregulation-based CPP targets is essential for giving this strategy a chance to demonstrate its effectiveness, should such management approaches be pursued. This aspect might need to be considered when designing clinical protocols or trial protocols for such approaches: options might include a higher frequency of CPP target reviews or the implementation of automated alerts when CPPopt time trend dynamics vary significantly.

Targeting CPP at CPPopt always improves cerebrovascular autoregulation, but the effect is tangible only when there exists some margin for improvement. To account for this aspect, in our analysis, we performed a group comparison at different levels of the best achievable PRx. The groups were represented by the control and intervention arms of the COGiTATE trial. In the control group of the trial, autoregulation was not considered when CPP targets were set. The best achievable PRx or PRxopt is the pressure reactivity value that corresponds to the CPPopt. Our results show that in the intervention arm of the trial, as opposed to the control arm, PRx was closer to PRxopt when PRxopt was more negative and in a range that indicated preserved autoregulation (see Table 2). In these periods, which cumulatively represented half of the monitoring periods with CPPopt available in our cohort, the curve that describes the relationship between PRx and CPP would normally be spanning from impaired to preserved vascular reactivity for different values of CPP (see the left example of Fig. 1). These are the periods when targeting CPPopt has the potential not only to improve vascular reactivity but also to bring it to a functioning state. When PRxopt was higher and not in a range of preserved autoregulation, the whole curve would also be in that range. In those cases, factors other than CPPopt should be considered. Hence, the main message of our results is the necessity of considering the shape and the location of the curve that describes the relationship between PRx and CPP when adopting autoregulation-guided management in the clinical setting (see Fig. 1).

Our results also suggest that the simple comparison of PRx average values between the control and intervention groups would not provide the information required for the assessment of the effectiveness of autoregulation-guided management in TBI. These concepts were also explored in a retrospective analysis by Svedung Wettervik et al. [16], in which the authors demonstrated that in a cohort of 383 patients with TBI, a higher percentage of monitoring time with both CPP close to CPPopt and low PRx was associated with favorable outcome. However, the authors found a similar strength of outcome associations for both PRx levels (the higher PRx, the stronger the association with unfavorable outcome) as well as deviation of CPP from CPPopt levels (favorable outcome was associated with lower deviation of CPP from CPPopt). The authors concluded that both approaches have their merits and they might represent different solutions for individualized management of CPP depending on the physiological scenario.

### Limitations

Our study is a secondary analysis on data collected for the COGiTATE trial. The sample was dictated by the main trial inclusion numbers. The COGiTATE trial was not powered for physiological outcome analysis. The implementation of our results in the clinical environment should await confirmation as the primary outcome in an adequately powered study. The current limitations and benefits of using PRx and CPPopt, as well as the steps required to achieve a broader application of PRx and CPPopt in clinical practice, have been discussed extensively in prior publications [3, 17–19].

## Conclusions

Our study presents findings that support the fact that CPPopt does provide a way of improving cerebrovascular reactivity in patients with TBI. We also bring insight into aspects of the PRx/CPP relationship (i.e., shape of the curve) that should be considered for autoregulation-guided management for future clinical protocols and trials design.

## Data Availability

The data sets analyzed during the current study are available from the corresponding author on reasonable request.

## Code Availability

The R and python codes used for this study are not publicly available but may be made available on reasonable request to the corresponding author.
